# Complete genome analysis of pathogenic *Metschnikowia bicuspidata* strain MQ2101 isolated from diseased ridgetail white prawn, *Exopalaemon carinicauda*

**DOI:** 10.1186/s12866-023-02865-2

**Published:** 2023-04-29

**Authors:** Wen-jun Shi, Ran Zhao, Jian-qiang Zhu, Xi-he Wan, Li-bao Wang, Hui Li, Song Qin

**Affiliations:** 1 Key Laboratory of Biology and Bioresource Utilization, Yantai Institute of Coastal Zone Research, Chinese Academy of Sciences, No. 17, Chunhui Road, Yantai, Shandong Province 264003 People’s Republic of China; 2 Institute of Oceanology & Marine Fisheries, No. 31, Jiaoyu Road, Nantong, Jiangsu 226007 People’s Republic of China; 3 University of Chinese Academy of Sciences, Beijing, 100049 People’s Republic of China; 4 National Demonstration Center for Experimental Fisheries Science Education, Shanghai Ocean University, Shanghai, 201306 People’s Republic of China

**Keywords:** *Exopalaemon carinicauda*, *Metschnikowia bicuspidata*, Complete genome sequence, Virulence genes, Metabolic mechanism

## Abstract

**Background:**

*Metschnikowia bicuspidata* is a pathogenic yesst that can cause disease in many different economic aquatic animal species. In recent years, there was a new disease outbreak in ridgetail white prawn (*Exopalaemon carinicauda*) in coastal areas of Jiangsu Province China that was referred to as zombie disease by local farmers. The pathogen was first isolated and identified as* M. bicuspidata*. Although the pathogenicity and pathogenesis of this pathogen in other animals have been reported in some previous studies, research on its molecular mechanisms is still very limited. Therefore, a genome-wide study is necessary to better understand the physiological and pathogenic mechanisms of *M. bicuspidata*.

**Result:**

In this study, we obtained a pathogenic strain, MQ2101, of *M. bicuspidata* from diseased *E. carinicauda* and sequenced its whole genome. The size of the whole genome was 15.98 Mb, and it was assembled into 5 scaffolds. The genome contained 3934 coding genes, among which 3899 genes with biological functions were annotated in multiple underlying databases. In KOG database, 2627 genes were annotated, which were categorized into 25 classes including general function prediction only, posttranslational modification, protein turnover, chaperones, and signal transduction mechanisms. In KEGG database, 2493 genes were annotated, which were categorized into five classes, including cellular processes, environmental information processing, genetic information processing, metabolism and organismal systems. In GO database, 2893 genes were annotated, which were mainly classified in cell, cell part, cellular processes and metabolic processes. There were 1055 genes annotated in the PHI database, accounting for 26.81% of the total genome, among which 5 genes were directly related to pathogenicity (identity ≥ 50%), including hsp90, PacC, and PHO84. There were also some genes related to the activity of the yeast itself that could be targeted by antiyeast drugs. Analysis based on the DFVF database showed that strain MQ2101 contained 235 potential virulence genes. BLAST searches in the CAZy database showed that strain MQ2101 may have a more complex carbohydrate metabolism system than other yeasts of the same family. In addition, two gene clusters and 168 putative secretory proteins were predicted in strain MQ2101, and functional analysis showed that some of the secretory proteins may be directly involved in the pathogenesis of the strain. Gene family analysis with five other yeasts revealed that strain MQ2101 has 245 unique gene families, including 274 genes involved in pathogenicity that could serve as potential targets.

**Conclusion:**

Genome-wide analysis elucidated the pathogenicity-associated genes of *M. bicuspidate* while also revealing a complex metabolic mechanism and providing putative targets of action for the development of antiyeast drugs for this pathogen. The obtained whole-genome sequencing data provide an important theoretical basis for transcriptomic, proteomic and metabolic studies of *M. bicuspidata* and lay a foundation for defining its specific mechanism of host infestation.

**Supplementary Information:**

The online version contains supplementary material available at 10.1186/s12866-023-02865-2.

## Background

The ridgetail white prawn, *Exopalaemon carinicauda,* is an important commercial shrimp species that is naturally distributed along the coasts of the Yellow Sea and the Bohai Sea in China and possesses multiple merits, such as high reproductive ability, a fast growth rate, wide environmental adaptability and a short reproductive cycle [1, 2]. With the continuous development of intensive aquaculture, deterioration in the ecological environment and germplasms have begun to appear, and environmental stressors, highly toxic bacteria and viruses have led to the frequent occurrence of shrimp diseases in recent years [3]. For example, milky shrimp disease caused by *Hematodinium* [4], red body disease caused by *Vibrio harveyi* [5]. and white spot syndrome caused by white spot syndrome virus (WSSV) [6]. have greatly hampered the development of the ridgetail white prawn culture industry.

*Metschnikowia bicuspidata* was first isolated from diseased *Daphnia magna* by Metchnikoff in 1884 [7]. Subsequently, infections attributed to this yeast were recorded in various aquatic animals, including *Macrobrachium rosenbergii* [8], *Daphnia dentifera* [9], *Portunus trituberculatus* [10], and *Eriocheir sinensis* [11]. Early studies have shown that *M. bicuspidata* is capable of spreading the infection to other organisms through specific vectors. For example, adult *Artemia* shrimp are one of the best foods for culturing fish fry, and when *M. bicuspidata* infects *Artemia*, the shrimp can act as a vector for the transmission of infection to chinook salmon [12]. In recent years, there have been an increasing number of reports about the infection of economically valuable aquatic animals by *M. bicuspidata*. In 2018, cases of pathogenic *M. bicuspidata* infection were found in Chinese mitten crabs in Liaoning, China, in which the diseased crabs accumulated milky white fluid, their activity was reduced, and their walking limbs were easily dislodged, resulting in crab mortality with an infection rate greater than 20% [11]. In 2018–2021, a new epidemic known as zombie disease occurred in some ridgetail white prawn farms in the coastal areas of Jiangsu Province, China, resulting in a decline in production and significant economic losses, and the pathogen was identified as *M. bicuspidata*.

Chen et al. proposed that *M. bicuspidata* present in pond water or sediment infects shrimp through contact with their mouth or gills; then, it is engulfed by the host haemolymph and encapsulated in membranous vesicles, which remain viable under limited replication conditions. When a decreased water temperature leads to increased sensitivity of the host to yeast, yeast multiplies rapidly in haemolymph, which eventually releases yeast cells in large numbers, leading to shrimp mortality [13]. Moore et al. suggested that *M. bicuspidata* actively infests the intestine of salmon by releasing needle-like ascospores via dehiscence, and the needle-tip morphology confers ascospores with the ability to penetrate inner layers of the host intestine, which allows yeast to propagate the infection from the intestine and eventually spread throughout the body [12]. Although *M. bicuspidata* has been reported many times worldwide, studies of its molecular pathogenesis are still very limited. Furthermore, owing to the lack of genomic information, it is extremely difficult to conduct molecular studies and the functional characterization of genes in *M. bicuspidata*.

In the present study, strain MQ2101 of *M. bicuspidata* was isolated from diseased *E. carinicauda,* and its pathogenicity to shrimp was verified by artificial infection experiments. Subsequently, the whole genome of strain MQ2101 was sequenced, assembled, annotated and analysed in detail. Our findings provide a basis for further research on the molecular mechanisms of pathogenicity and metabolism in this yeast and the development of strategies to prevent the spread of infection.

## Results and discussion

### Pathogenicity of strain MQ2101

Diseased *E. carinicauda* naturally infected by strain MQ2101 showed classic symptoms, including a reddish body colour, whitish muscles, enlarged appendages and slow swimming. When the carapace was opened, large amounts of a milky effusion flowed out. The hepatopancreas exhibited erosion, and muscles were liquefied to varying degrees, showing opaque white colour. The colour of the haemolymph also changed from its normal transparent state to milky white. The morbidity and mortality rates caused by this infection in different aquaculture models were 5%-30% and 3%-10%, respectively.

**Fig. 1:**
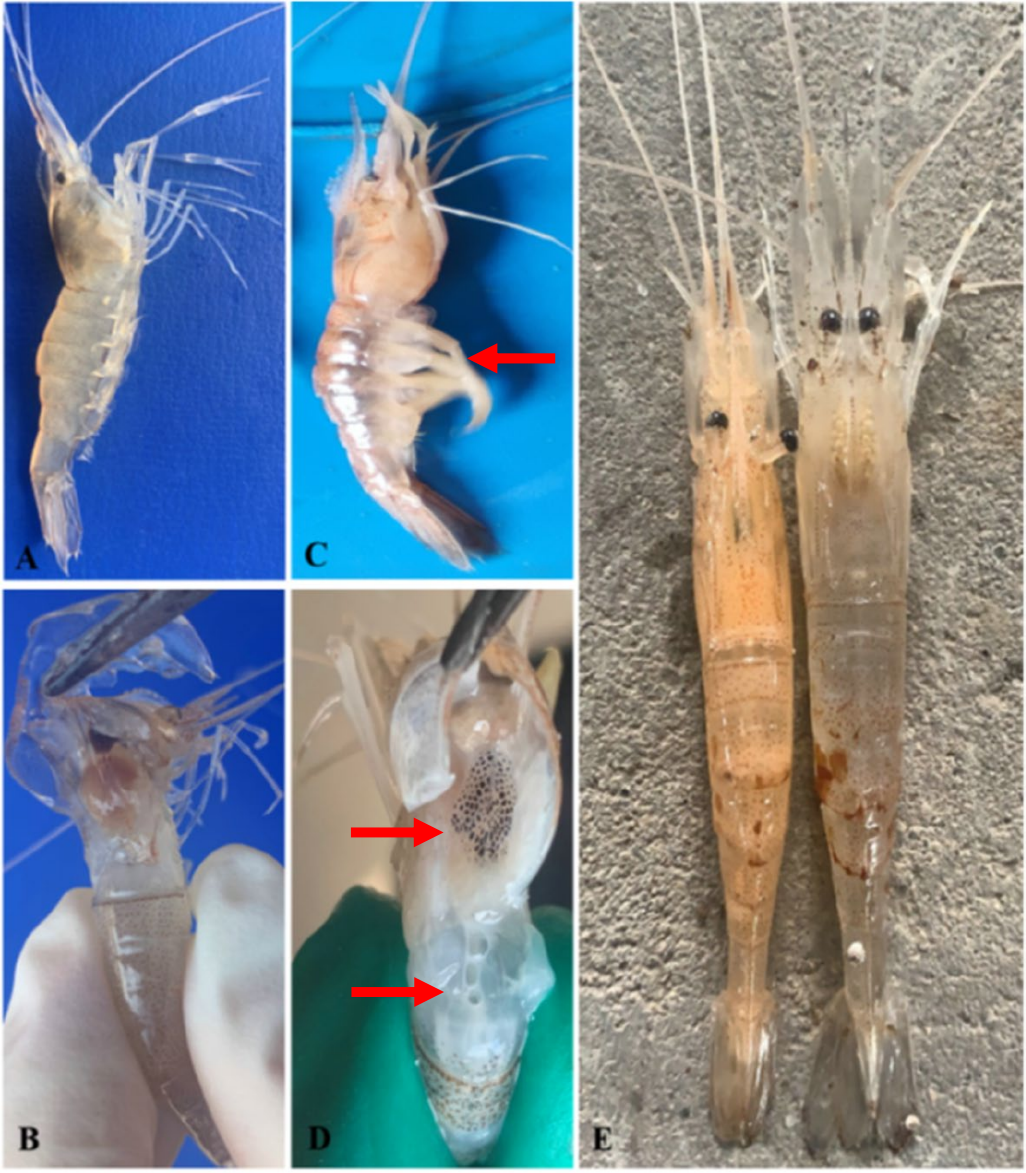
Comparison of healthy and artificially infected *E. carinicauda* of *M. bicuspidata* strain MQ2101. A,B are healthy *E. carinicauda*. C, D are naturally diseased *E. carinicauda* with swollen white appendages, hepatopancreas erosion, and milky liquid under the carapace. E is the artificially infected *E. carinicauda* with strain MQ2101 (left) and normal saline (right). The red arrows indicate infected areas

During the artificial challenge test, the shrimp in the negative control group treated with normal saline remained healthy and showed no symptoms. The shrimp that were artificially infected with strain MQ2101in the experimental group showed the same symptoms as the naturally infected shrimp, such as reddish body colour, milky liquid under the carapace, pale appendages and swelling. Strain MQ2101 was also isolated and identified from artificially infected shrimp. Artificial infection confirmed that *M. bicuspidata* strain MQ2101 showed high pathogenicity in shrimp. Figure 1 shows the results of artificial infection.

### Genome sequencing and general features of strain MQ2101

**Fig. 2:**
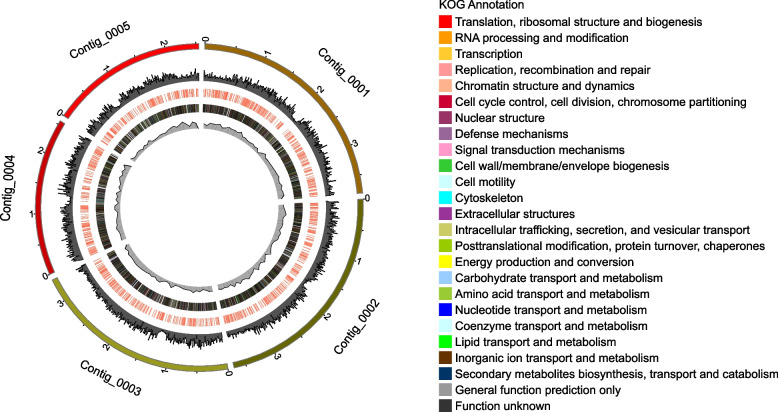
Circular genome maps of *M. bicuspidata* strain MQ2101. Note: The outermost circle is each segment of the genome. The second circle is the GC content. The third circle shows PHI annotations, with annotated genes marked in red. The fourth circle is the annotation of the gene with KOG annotation and the legend of KOG annotation is attached on the right side of the picture. The innermost circle is the GC-Skew value

The genome of *M. bicuspidata* strain MQ2101 was sequenced using a third-generation sequencing approach. A total of 7.1 Gb of raw sequences were generated from the Illumina sequencing platform by Genedenovo Biotechnology Co., Ltd. (Guangzhou, China). After assembly, as shown in Table 1, the size of the whole genome of *M. bicuspidata* strain MQ2101 was 15.98 Mb, and it was assembled into 5 scaffolds with an approximate GC content of 47.8%. The key assembly parameter N50 was 3.3 Mb. The whole genome encoded 3934 predicted open reading frames (ORFs). The size category > 3,000 bp included the largest number of genes. With regard to noncoding genes, we identified 250 transfer RNAs (tRNAs) and 86 ribosomal RNAs (rRNAs) from the assembly. Supplementary Figure S1 illustrates the gene length distribution of *M. bicuspidata* strain MQ2101. Figure 2 shows the genome information.

**Table 1:** Genome features of *M. bicuspidata* strain MQ2101

Features	MQ2101
Scaffolds	5
Size(Mb)	15.98
GC content(%)	47.87
N50 (Mb)	3.38
Protein-coding genes	3934
Average gene length (bp)	1474
tRNA	250
rRNA	86

Repeated DNA sequences and transposable elements play important roles in fungal evolution, genome structure and gene function [14]. The *M. bicuspidata* strain MQ2101 genome comprised 4.70% repetitive DNA and 10.92% transposable elements. The transposable elements were mainly classified into 15 families. Most of them were retrotransposons, with particularly high abundance of the DIRS (447), LTR (331), and LINE (132) subclasses (Table 2).

**Table 2:** Repeated sequences in *M. bicuspidata* strain MQ2101

Family name	Class	Number
Sola	Class I	5
Novosib	Class I	1
Kolobok	Class I	1
IS3EU	Class I	1
Dada	Class I	6
Academ	Class I	4
Maverick	Class I	10
Helitron	Class I	115
Crypton	Class I	7
TIR	Class I	168
Unknow	Class I	1
SINE	Class II	6
DIRS	Class II	447
LINE	Class II	132
LTR	Class II	447

Class I DNA transposons; Class II retrotransposons

### Phylogenetic analyses

**Fig. 3:**
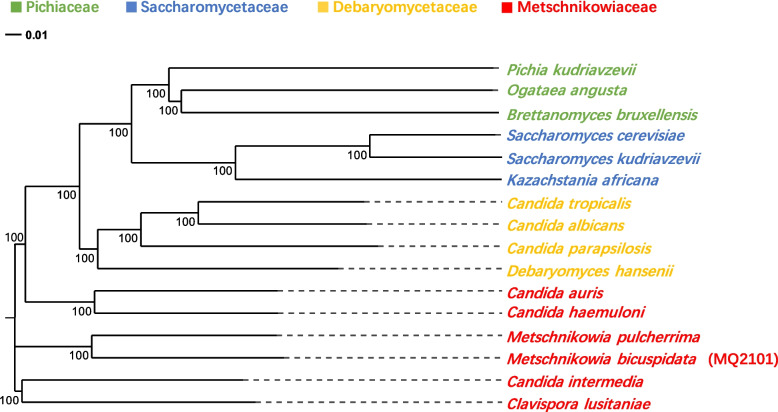
Phylogenetic relationship of *M. bicuspidata* strain MQ2101 and the complete genomes of fifteen bacterial strains downloaded from the NCBI database

Evolutionary trees are used in biology to represent the evolutionary relationships between species and provide insight for the exploration of fungal evolutionary processes. In this study, we selected 15 different Saccharomycetes along with strain *M. bicuspidata* strain MQ2101 for the construction of an evolutionary tree, the selection criteria were the yeasts that have reported pathogenicity and released genomic information, belonging to four different families of the same order as *Metschnikowia bicuspidate*. The phylogenetic analysis revealed the evolutionary relationships among yeasts taxa and the positioning of *M. bicuspidata* strain MQ2101 within *Metschnikowiaceae* (Fig. 3).

### Genome functional annotation

A total of 3934 protein-coding genes were predicted in the whole genome of strain MQ2101. Among these genes, 3899 genes with biological functions were annotated in several basic databases. Only 3051 (77.55%), 2627 (66.78%), 2493 (63.37%), and 3899 (99.11%) of the predicted genes showed homology with known functions in the SwissProt, Clusters of Orthologous Groups (COG)/Eukaryotic Orthologous Groups (KOG), Kyoto Encyclopaedia of Genes and Genomes (KEGG), and Nonredundant protein (NR) databases, respectively. There were 2095 genes common to the results obtained from all of these classical databases. Supplementary Figure S2 shows the genes annotated in the COG/KOG, KEGG, NR and SwissProt databases.

**Fig. 4:**
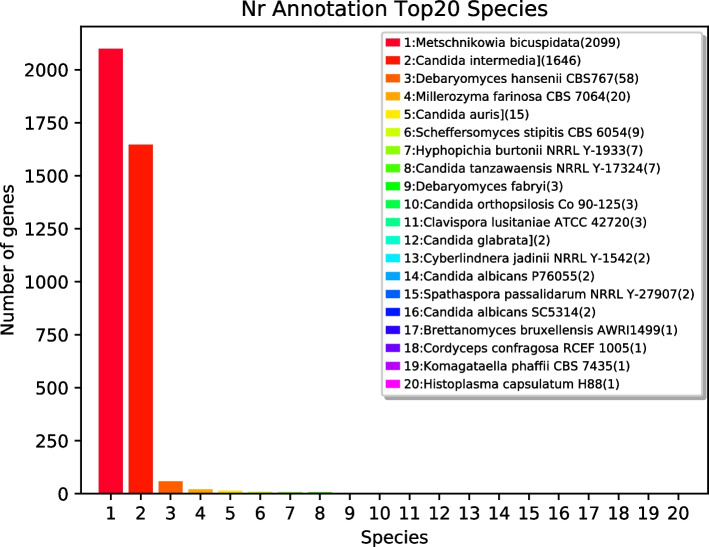
NR annotation analysis of *M. bicuspidata* strain MQ2101

Homologous sequences were annotated in the NR database. The species with the highest homology to *M. bicuspidata* strain MQ2101 was *Metschnikowia bicuspidate* (53.83%), followed by *Candida intermedia* (42.22%), *Debaryomyces hansenii* CBS767 (1.49%), *Millerozyma farinosa* CBS 7064 (0.51%), *Candida auris* (0.38%) and *Scheffersomyces stipitis* CBS 6054 (0.23%) (Fig. 4). Supplementary Table S1 lists the Nr annotation information.

**Fig. 5:**
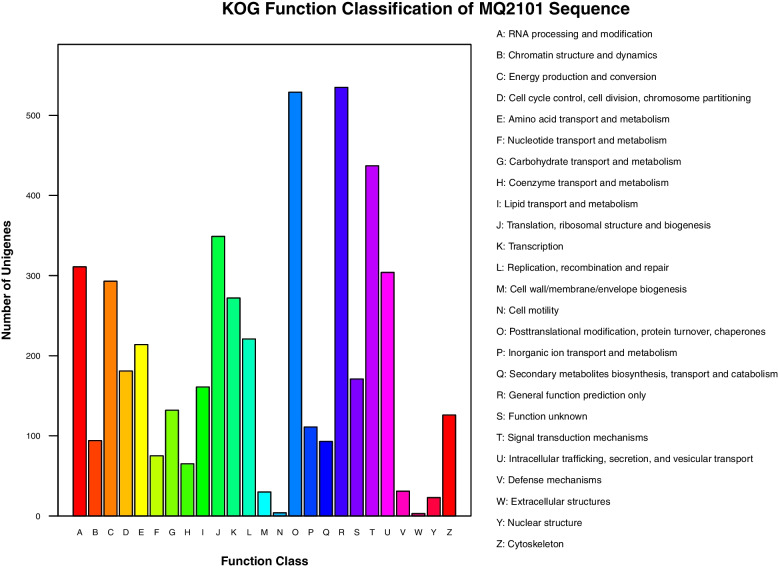
KOG functional annotation of *M. bicuspidata* strain MQ2101

KOG analysis was performed to elucidate the possible biological functions and mechanisms of annotated genes in *M. bicuspidata* strain MQ2101. The predicted functional ORFs were categorized into 25 classes based on KOG categorization, which contain R (535 ORFs, General function prediction only), O (529 ORFs, Posttranslational modification, protein turnover, chaperones), T (437 ORFs, Signal transduction mechanisms), J (349 ORFs, Translation, ribosomal structure and biogenesis), A (311 ORF, RNA processing and modification), U (304 ORFs, Intracellular tra cking, secretion, and vesicular transport), C (293 ORFs, Energy production and conversion), K (272 ORFs, Transcription), L (221 ORFs, Replication, recombination and repair), E (214 ORFs, Amino acid transport and metabolism), D (181 ORFs, Cell cycle control, cell division, chromosome partitioning), S (171 ORFs, Function unknown), I (161 ORFs, Lipid transport and metabolism), G (132 ORFs, Carbohydrate transport and metabolism), Z (126 ORFs, Cytoskeleton), P (111 ORFs, Inorganic ion transport and metabolism), B (94 ORF, Chromatin structure and dynamics), Q (93 ORFs, Secondary metabolites biosynthesis, transport and catabolism), F (75 ORFs, Nucleotide transport and metabolism), H (65 ORFs, Coenzyme transport and metabolism),V (31 ORFs, Defense mechanisms), M (30 ORFs, Cell wall/membrane/envelope biogenesis), Y (23 ORFs, Nuclear structure), N (4 ORFs, Cell motility), W (3 ORFs, Extracellular structures) (Fig. 5). Supplementary Table S2 lists the KOG annotation information.

**Fig. 6:**
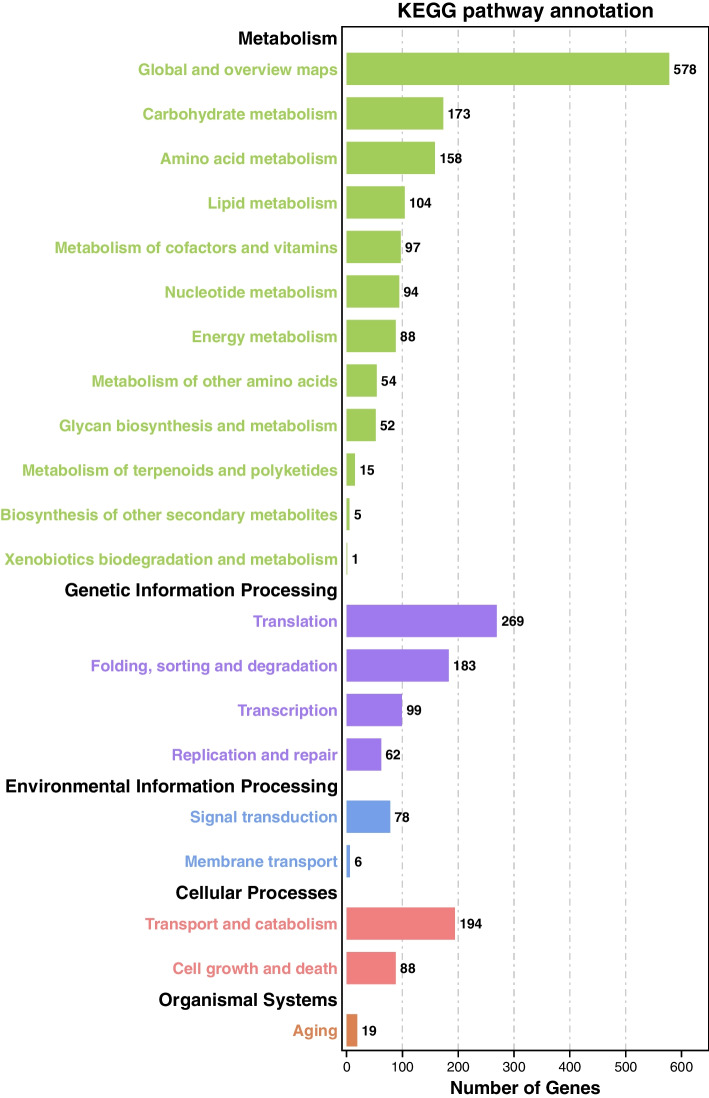
KEGG annotation of *M. bicuspidata* strain MQ2101 genome

Alternatively, 2493 genes in *M*. *bicuspidata* strain MQ2101 were categorized into five classes based on KEGG categorization, including cellular processes, environmental information processing, genetic information processing, metabolism and organismal systems. The genes in the cellular process classification were divided into 2 major categories, where the most-enriched cluster corresponded to transport and catabolism (194 genes), followed by cell growth and death (88 genes). The genes in the classification of environmental information processing can be divided into 2 categories, where the most-enriched cluster corresponded to signal transduction (78 genes), followed by membrane transport (6 genes). In the genetic information processing classification, genes were classified into four categories, where the most-enriched cluster corresponded to translation (269 genes) and folding, sorting and degradation (183 genes). In the metabolism classification, genes were classified into 12 categories, where the most-enriched cluster corresponded to global and overview maps (578 genes), carbohydrate metabolism (173 genes) and amino acid metabolism (158 genes). In the organismal systems classification, there was only one gene cluster, corresponding to ageing (19 genes) (Fig. 6). Supplementary Table S3 lists the KEGG annotation information.

**Fig. 7:**
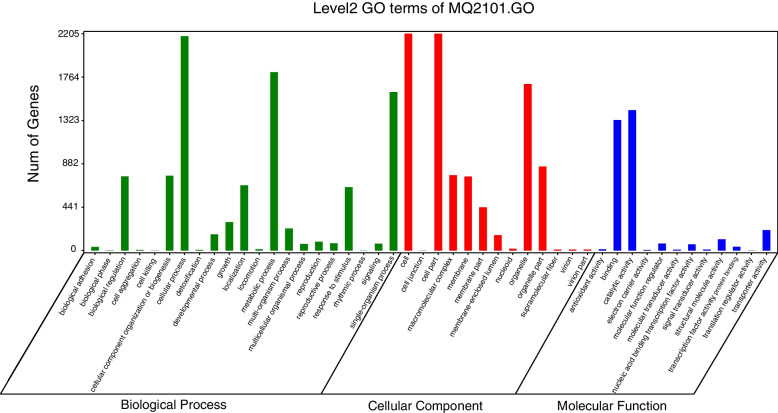
GO annotation of *M. bicuspidata* strain MQ2101 genome

In addition, GO database annotation results showed that 2893 genes were primarily involved in biological processes, cellular components and molecular functions, accounting for 73.54% of the total genes in *M*. *bicuspidata* strain MQ2101. In the biological process category, the genes were clustered into 21 classifications. In these classifications, the largest number of genes were involved in cellular processes (2178 genes, 55.36%), followed by metabolic processes (1812 genes, 46.06%) and single-organism processes (1611 genes, 40.95%). In the cellular component classification, the annotated genes were clustered into 13 categories, and the most annotated components in these classifications were cell (2205 genes, 56.05%) and cell part (2205 genes, 56.05%), followed by organelle (1692 genes, 43.01%) and organelle part (854 genes, 21.71%). Among the molecular functions, the annotated genes were clustered into 12 categories, with the largest number of genes clustering in the catalytic activity category (1426 genes, 36.25%), followed by binding (1328 genes, 33.76%) and transporter activity (207 genes, 5.26%) (Fig. 7). Supplementary Table S4 lists the GO annotation information.

### Prediction of virulence genes of strain MQ2101

**Fig. 8:**
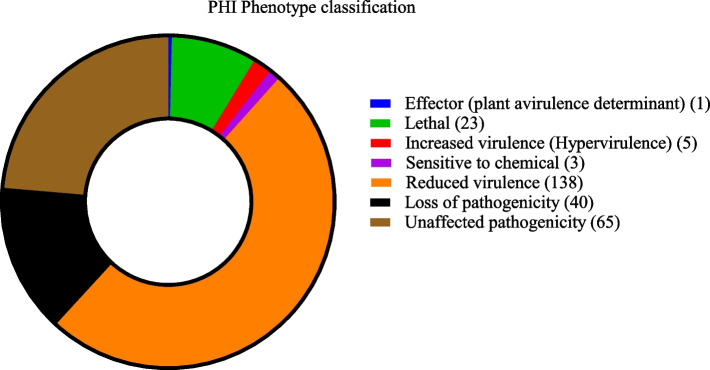
PHI classification result of *M. bicuspidata* strain MQ2101 genome

To date, there have been few studies on virulence factors of *M. bicuspidata*, and no specific virulence genes have been reported. To identify genes involved in pathogenicity in *M. bicuspidata* strain MQ2101, a BLAST search against the pathogen‒host interaction (PHI) gene database was conducted. A total of 1055 genes had annotation information in the PHI databases, and 275 genes showed identity ≥ 50%. Among these genes, 23 genes belonged to lethal factors, 5 genes were related to hypervirulence, 3 genes were related to drug resistance, 1 gene was related to effectors, and 243 genes were unrelated to pathogenicity or caused reduced virulence of pathogenic bacteria (Fig. 8). Supplementary Table S5 lists the PHI annotation information.

The products of lethal factor genes, although not directly involved in fungal pathogenesis, play an essential role in fungal survival and could be used as antifungal targets for future research to improve drugs to prevent related diseases. The lethal factors of strain MQ2101 included protein kinase, saccharopine dehydrogenase, transcription factor, 3'-phospho-adenylylsulfate reductase, tryptophan synthase, β-1,3-glucan, and glutamate-tRNA synthase proteins. The synthesis of β-1,3-glucan, the major structural component of the yeast cell wall, is synchronized with the budding cycle [15]. The inhibition of β-1,3-glucan synthase activity can inhibit fungal growth and reproduction, making glucan synthase an ideal target for antifungal drugs [16, 17]. A variety of protein kinases from *M. bicuspidata* have also been identified in the PHI database. Protein kinases (PKs) are involved in the regulation of various growth and developmental processes and responses to environmental stimuli in eukaryotic organisms [18] and could be potential targets for drug research. Hypervirulence genes are vital genes associated with pathogenicity. The hypervirulence genes of strain MQ2101 included heat shock protein, HSP90, pH signalling transcription factor, PacC, cell-wall integrity, mkkA, rapamycin sensitivity, BcFKBP12, phosphate acquisition and storage, and PHO84 genes. The activities of many metastable signal transducers involved in detecting and reacting to stress are regulated by the conserved molecular chaperone Hsp90 in eukaryotes [19, 20]. Hsp90 controls fungal survival, drug resistance, and virulence in a variety of pathogenic fungi [21, 22]. PacC is essential for the synthesis of enzymes and metabolites as well as for the remodeling of the fungal cell wall, all of which are necessary for infection [23]. By constructing and studying PacC deletion strains of a variety of fungi, this regulatory factor was found to be required for the virulence of human, insect and plant pathogens [24–27]. PHO84 mainly regulates phosphate input at the fungal cell surface, and we found that the loss of *C. albicans* PHO84 attenuated virulence in Drosophila and mice [28].

The DFVF database provides a list of known fungal virulence issues. It includes 2058 pathogenic genes that were made available by 228 fungal strains from 85 genera [29]. In the current genomic investigation, DFVF included 235 genes, or 5.97% of the anticipated genes. G00554 (95.735%), G00069 (94.231%), G02619 (93.333%), G02867 (93.023%), G02939 (92.248%), G01063 (90.661%), G03488 (90.582%), G03776 (90.341%), and G03239 (90.127%) were the genes with identity values greater than 90. The specific functional annotations of these nine genes in the DFVF database are shown in Table 3. All of the above pathogenicity-related genes are potential virulence factors in the *M. bicuspidata* genome. Supplementary Table S6 lists the DFVF annotation information.

**Table 3:** Gene product name and functions of the top 9 genes in DFVF

Gene ID/	Functions
G00554	Unknown
G00069	FUNCTION: Involved in hyphal formation, virulence, morphogenesis.//SUBCELLULAR LOCATION: Cell membrane; Lipid-anchor; Cytoplasmic side (Potential).//SIMILARITY: Belongs to the small GTPase superfamily. Rho family. CDC42 subfamily
G02619	FUNCTION: Tubulin is the major constituent of microtubules. It binds two moles of GTP, one at an exchangeable site on the beta chain and one at a non-exchangeable site on the alpha-chain (By similarity).//SUBUNIT: Dimer of alpha and beta chains (By similarity).//SIMILARITY: Belongs to the tubulin family
G02867	SIMILARITY: Belongs to the small GTPase superfamily. Rab family
G02939	SIMILARITY: Belongs to the 14–3-3 family
G01063	Unknown
G03488	SIMILARITY: Belongs to the actin family
G03776	FUNCTION: Mitogen-activated protein kinase involved in a signal transduction pathway that is activated by changes in the osmolarity of the extracellular environment. Controls osmotic regulation of transcription of target genes. Regulates stress- induced production and accumulation of glycerol and D-arabitol. HOG1 is also involved in virulence, morphogenesis and oxidative stress response especially through its role in chlamydospore formation, an oxygen-dependent morphogenetic program.//CATALYTIC ACTIVITY: ATP + a protein = ADP + a phosphoprotein.//COFACTOR: Magnesium (By similarity).//ENZYME REGULATION: Activated by tyrosine and threonine phosphorylation (By similarity).//SUBCELLULAR LOCATION: Cytoplasm. Nucleus. Note = Predominantly cytoplasmic in unstressed cells but rapidly concentrates within the nucleus in response to hyperosmotic conditions and phosphorylation.//DOMAIN: The TXY motif contains the threonine and tyrosine residues whose phosphorylation activates the MAP kinases.//PTM: Dually phosphorylated on Thr-174 and Tyr-176, which activates the enzyme (By similarity). Phosphorylated in response to oxidative and salt stress.//SIMILARITY: Belongs to the protein kinase superfamily. Ser/Thr protein kinase family. MAP kinase subfamily. HOG1 sub-subfamily.//SIMILARITY: Contains 1 protein kinase domain
G03239	CATALYTIC ACTIVITY: A phosphoprotein + H(2)O = a protein + phosphate.//SIMILARITY: Belongs to the PPP phosphatase family.//CAUTION: The sequence shown here is derived from an EMBL/GenBank/DDBJ whole genome shotgun (WGS) entry which is preliminary data

### CAZymes

**Fig. 9:**
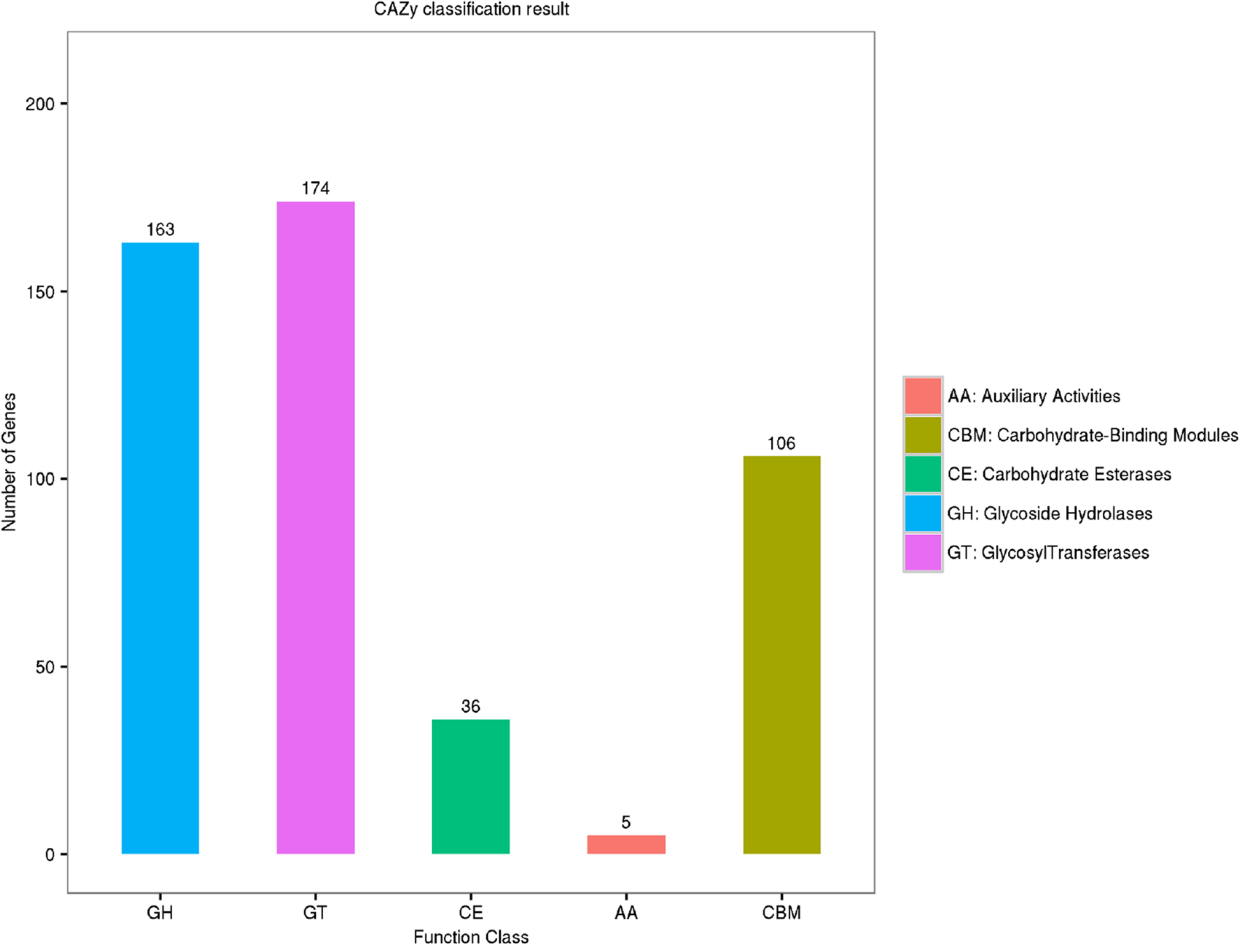
CAZy classification result of *M. bicuspidata* strain MQ2101 genome

Carbohydrate-active enzymes (CAZymes) are related enzyme families that can catalyse carbohydrate degradation, modification and biosynthesis and play an important role in a series of biological processes in fungi. The main categories of CAZymes are glycoside hydrolases (GHs), carbohydrate esterases (CEs), glycosyl transferases (GTs), and auxiliary activities (AAs). In addition, carbohydrate-binding modules (CBMs) are also included [30]. CAZymes are involved in the degradation of cell walls and storage compounds in many plant-pathogenic fungi and play an integral role in the infestation process [31, 32]. In contrast, CAZymes in animal-pathogenic fungi have been studied mainly in certain insect-pathogenic fungi, which also play an indispensable role in the pathogenic process [33]. A total of 484 CAZyme-encoding gene homologues were identified by comparing the genomic data of strain MQ2101 with the CAZy database. GTs accounted for the largest proportion (35.95%) of these CAZyme families, followed by GHs (33.67%), and then CBMs (21.9%). Few genes encoded CEs and AAs, accounting for 7.43% and 1.03% of the predicted genes, respectively (Fig. 9). GTs are key enzymes that catalyse the formation of glycosidic bonds by assisting in the transfer of sugar residues from donors to specific acceptor molecules [34, 35]. GHs are a class of enzymes that catalyse the hydrolysis of glycosidic bonds and are primarily responsible for the degradation of carbohydrates [36]. CBMs are noncatalytic structural domains typically attached to carbohydrate-active enzymes, with a broad range of conserved structures that recognize a wide variety of carbohydrates (both soluble and insoluble) [37]. This series of CAZymes play a key role in the life course of *M. bicuspidata*. This study also compared the quantitative differences between the CAZymes of *M. bicuspidata* and five other yeasts from the same family and showed that their levels were significantly higher in *M. bicuspidata* than in the other five yeasts (Table 4), which may suggest unique physiological characteristics and infestation mechanisms in *M. bicuspidata* strain MQ2101. Supplementary Table S7 lists the CAZy database annotation information.

**Table 4:** Comparative analysis of CAZyme family among various pathogen fungi

Species	GHs	GTs	CEs	AAs	CBMs	Total
MQ2101	163	174	36	5	106	484
*Metschnikowia aff. pulcherrima*	58	68	2	4	9	141
*Candida auris*	55	75	2	6	10	148
*Candida intermedia*	69	76	2	6	9	162
*Candida haemuloni*	59	67	2	7	8	143
*Clavispora lusitaniae*	58	62	2	8	8	138

### Gene clusters of secondary metabolites

**Fig. 10:**
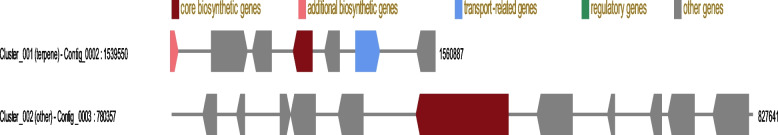
Putative gene clusters in *M. bicuspidata* strain MQ2101

Some secondary metabolites have been suggested to play essential roles in fungal infection [38]. The gene clusters related to secondary metabolites were predicted for strain MQ2101. Two gene clusters were identified, one of which was a terpene gene cluster, whereas the function of the other is not yet known (Fig. 10). The terpene gene cluster is composed of four unknown functional genes, two biosynthesis-related genes and one transport-related gene. G01372 is the core biosynthetic gene in this gene cluster, and functional analysis showed that this gene mainly encodes farnesyl-diphosphate farnesyltransferase, directing intermediates produced from mevalonate toward either the nonsterol pathway or the cholesterol synthetic pathway and playing a vital role in the cholesterol metabolic pathway [39]. G1369 is an additional biosynthetic gene encoding the Aldo/keto reductase of *M. bicuspidata*. The Aldo/keto reductase proteins are an NADP-dependent oxidoreductase superfamily whose members catalyse redox transformations involved in detoxification, intermediary metabolism and biosynthesis, among other physiological functions [40–42]. G01374 encodes the MFS general substrate transporter, a transport-related gene. MFS transporters selectively transport a wide range of substrates across membranes, are involved in a variety of physiological processes in organisms [43], and indirectly regulate the internal pH and stress response mechanisms in fungi [44]. There is now evidence that MFS transporters take part in multidrug resistance in fungi, as they are able to act as drug H^+^ antiporters in microorganisms [45]. The other gene cluster is composed of ten unknown functional genes and one biosynthesis-related gene. The core gene, LYS2, is primarily involved in the a-aminoadipate pathway for the biosynthesis of lysine [46]. The specific function of this gene cluster needs to be further investigated.

### Secreted protein prediction

It is necessary to elucidate the functions of yeast secretions to explore the pathogenic mechanisms of pathogenic yeast. By using siganlP4.1 to predict the signal peptides of gene sequences, the secretome of *M. bicuspidata* was predicted. In total, 163 putatively secreted proteins were identified from MQ2101, accounting for 4.14% of the total proteins in the genome. Among these proteins, 115 secreted proteins (70.55%) had clear functional annotations, which included amino acid transporters, aspartyl protease, cytochrome P450, ABC transporter, ferric reductase, multicopper oxidase, glycosyltransferase, and lysophospholipase proteins. The importance of cytochrome P450 and ABC transporters is emphasized by the fact that they often lead to multidrug resistance in microbial pathogens, thereby interfering with the effective treatment of infectious diseases [47–49]. Lysophospholipases and aspartyl proteases have been shown to be involved in the pathogenic processes of several pathogenic fungi [50–52]. These secreted proteins may play important roles during pathogenesis. Nearly 70% of the secreted proteins had functional annotations in the GO database, and the analysis showed that the top matched GO terms were cellular process and metabolic process in the biological process category, cell part in the cellular component category and catalytic activity and binding in the molecular function category. Supplementary Figure S3 illustrates the distribution of major secretory proteins in MQ2101.

### Gene family analysis

**Fig. 11:**
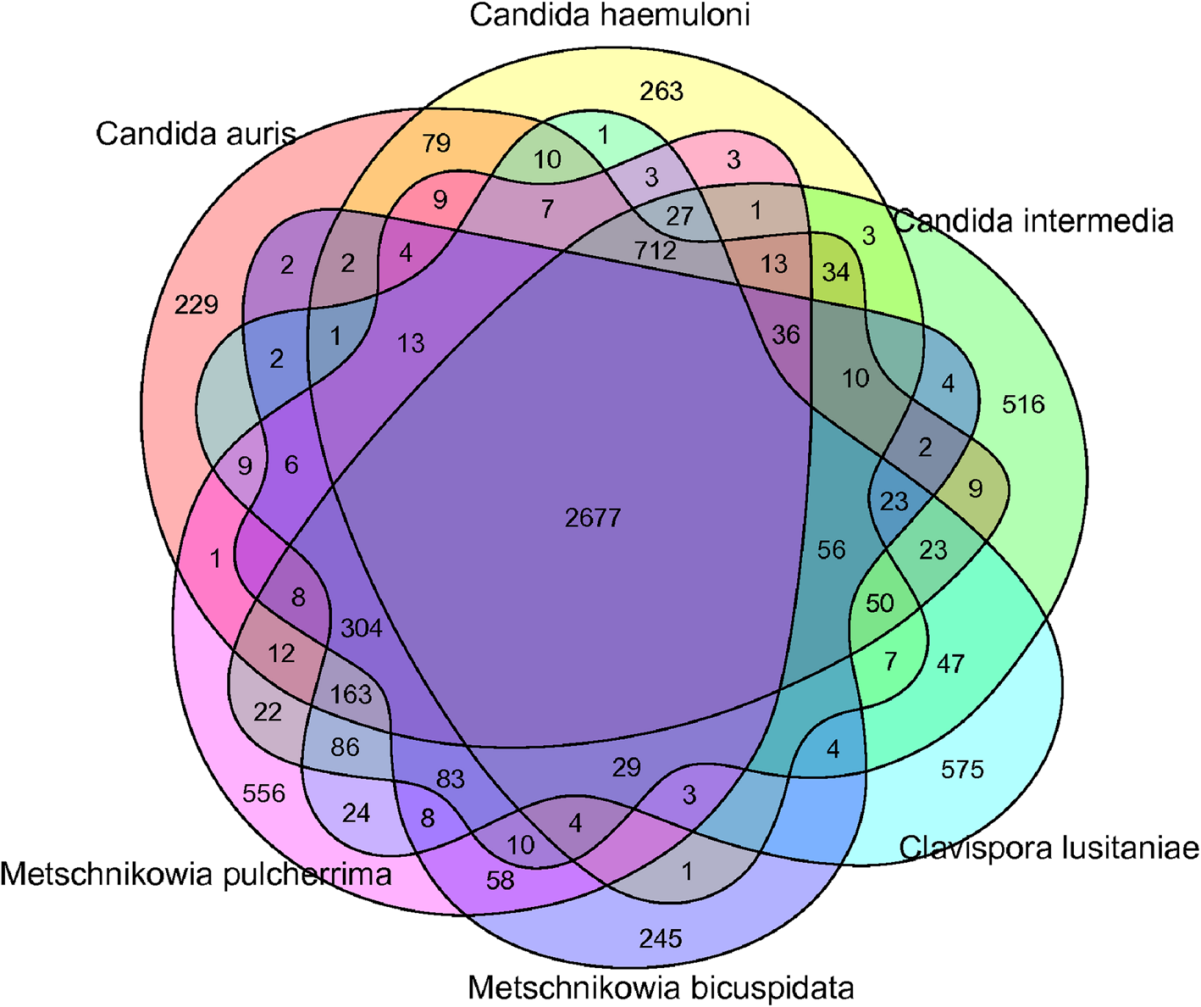
Common and specific gene families of *M. bicuspidata* strain MQ2101and five fungi

Gene family analysis facilitates the discovery of shared gene families of closely related yeasts and unique gene families that have evolved to adapt to different living environments. Based on the results of evolutionary tree analysis, the gene families of *M. bicuspidata*, *M. pulcherrima*, *C. intermedia*, *C. auris*, *C. lusitaniae* and *C. haemuloni* were subjected to cluster analysis. Cluster analysis revealed common and unique gene families in different yeasts. The numbers of total and unique gene families in the MQ2101 strain and other yeasts are presented in the form of a Venn diagram. The number of specific genes, the total number of gene families, and the number of genes unique to each family are shown in Fig. 11.

In total, 2,677 common gene families were identified among the six yeasts. *M*. *bicuspidata* contained members of 245 specific gene families, including 274 genes, among which 151 genes (55.1%) were annotated in the GO database. The analysis showed that 106 genes were involved in binding and 91 in catalytic activity, and these specific genes can serve as potential targets for modulating the pathogenicity of the strain. Supplementary Figure S4 illustrates the major distribution of specific genes in MQ2101.

## Conclusion

The whole genome of pathogenic *M. bicuspidata* strain MQ2101 with a size of 15.98 mb was annotated into 5 scaffolds. The genome contained 3934 coding genes, among which 3899 genes with biological functions were annotated in multiple underlying databases. There were 1055 genes annotated in the PHI database, accounting for 26.81% of the total genome, among which 5 genes were directly related to pathogenicity (identity ≥ 50%), such as hsp90, PacC, and PHO84. There were also some genes related to the activity of the yeast itself, which could be used targets for the development of antiyeast drugs. The analysis based on the DFVF database showed that strain MQ2101 contained 235 potential virulence genes. BLAST searches in the CAZy database showed that strain MQ2101 may have a more complex carbohydrate metabolism system than other yeasts of the same family. Two gene clusters and 168 putative secretory proteins were predicted in strain MQ2101, and functional analysis showed that some of the metabolic proteins may be directly involved in the pathogenicity of the strain. Gene family analysis with six closely related yeasts revealed that *M. bicuspidata* strain MQ2101 has 245 unique gene families comprising 274 genes, which could serve as potential targets involved in pathogenicity. Genome-wide analysis preliminarily explained the pathogenicity of *M. bicuspidata* while also revealing a complex metabolic mechanism and providing putative targets for the development of antiyeast drugs for this pathogen, which will contribute significantly to the prevention and treatment of diseases caused by *M. bicuspidata* to ensure the safety of susceptible aquatic economic animals. The whole-genome sequencing data provided an important theoretical basis for transcriptomic, proteomic and metabolic studies of *M. bicuspidata* and laid a foundation for defining its specific mechanism of host infestation.

## Materials and methods

### Isolation of pathogenic strains

*M. bicuspidata* strain MQ2101 was isolated from the haemolymph of diseased *E. carinicauda* cultured on an outdoor farm in the coastal areas of Qidong City, Jiangsu Province, China. They were anaesthetized on ice, and the body surface of typical diseased shrimp was wiped with 75% ethanol. Subsequently, the haemolymph of diseased shrimp was drawn with a sterile needle, mixed with sterile saline at 1:2 (v/v) and then streaked onto potato dextrose agar (PDA, Land Bridge, Beijing, China) medium plates under aseptic conditions, followed by incubation at 27 °C for 48 h. After incubation, colony morphology on the plates was observed. The single colony with the greatest number of morphologically identical bacteria was used as the dominant bacterium and inoculated onto the same medium to obtain a pure isolate. The pure isolate was stored at -80 ℃ and re-cultured for subsequent experiments.

The pathogenicity of the strains was determined using a manual intraperitoneal injection challenge method. The experimental shrimp (weight: 3.8 ± 0.31 g, length: 5.8 ± 0.2 cm) were collected from the Rudong base of the Institute of Oceanology & Marine Fisheries, Jiangsu, Nantong, China. The shrimps were temporarily reared with reference to the previous method of our research group [53]. In brief, prior to the challenge experiment, shrimp were raised in a 500 L clean barrel tank for one week at 18 ± 0.5 °C, 25 ppt (containing around 250 shrimp in 350 L marine water). Throughout the procedure, the tanks were continually aerated to keep the amount of dissolved oxygen (DO) above 6.0 mg/L. The shrimp were routinely fed a commercial diet procured from Nantong Haid Biotech Co., LTD. at 3% of their wet body weight once per day during the acclimation period. The siphon tube was used to quickly remove any leftover diet after feeding, discharged waste, or dead animals. Fresh marine water (18 ± 0.5 °C, 25 ppt, DO no less than 6.0 mg/L) was also added once day, replacing about 30–40% of the tank's water.

After acclimation, the healthy shrimp were randomly divided into six 100 L cylindrical tanks (30 shrimp each) containing 75 L of aerated seawater (18 ± 0.5 °C, salinity: 25 ppt, DO above 6.0 mg/L). Then, the six tanks were divided into two groups, including a negative control group and an experimental group. The shrimp of each experimental group were injected intramuscularly with 20 μL of bacterial suspension (2.0 × 10^8^ cells/mL), and each shrimp of the negative control group were injected intramuscularly with 20 μL of sterile saline. During the experiment, the feeding and water changing procedures were the same as in the acclimation stage.

When the shrimp in the experimental group showed symptoms, the above steps were repeated to isolate the dominant bacteria, which were then purified and identified using PDA medium. After the isolates were identified as an *M. bicuspidata* strain, its whole genome was sequenced on the Illumina HiSeq X platform.

After the artificial challenge experiment, all of the experimental shrimp were anaesthetized on ice and soaked in chlorine dioxide disinfectant.

### Genomic DNA preparation and sequencing

Genomic DNA from *M. bicuspidata* MQ2101 was extracted using Fungi genomic DNA extraction kits (Solarbio, Beijing, China) in accordance to the manufacturer’s instructions. DNA quality was assessed using Qubit (Thermo Fisher Scientific, Waltham, MA) and Nanodrop (Thermo Fisher Scientific, Waltham, MA) instruments accordingly. Then the qualified genomic DNA from *M. bicuspidata* MQ2101 was fragmented with G-tubes (Covaris, Woburn, MA, USA) and end repaired to prepare SMRTbell DNA template libraries (fragment size > 10 Kb selected by BluePippin system) according to the manufacturer’s specifications (PacBio, Menlo Park, CA). Library quality was detected by Qubit® 2.0 Fluorometer (Life Technologies, CA, USA), and the average fragment size was estimated on a Bioanalyzer 2100 (Agilent, Santa Clara, CA). SMRT sequencing was performed on a Pacific Biosciences Sequel sequencer (PacBio, Menlo Park, CA) according to standard protocols (MagBead Standard Seq v2 loading, 1 × 180 min movie) using P4-C2 chemistry.

### de novo genome assembly

By utilizing MECAT to align shorter reads from the same library, the continuous long reads acquired from SMRT sequencing were adjusted for random errors in the long seed reads (seed length threshold 6 Kb) [54]. In order to do de novo assembly utilizing MECAT and an overlap-layout-consensus (OLC) method, the resultant corrected, preassembled reads were used [55]. Assemblies did not employ quality values since SMRT sequencing exhibits relatively little quality variation among the reads [56].

### Genomic prediction

Open reading frames (ORFs) were predicted using GeneMark-ES [57], which is a well-studied gene-finding program used for prokaryotic genome annotation. Repetitive elements were identified by RepeatMasker [58]. The prediction of noncoding RNAs, such as rRNAs, were carried out using RNAmmer [59], and tRNAs were identified by tRNAscan-SE [60].

### Genome annotations

To annotate the constructed sequences, we used a number of complimentary strategies. The genes were annotated by alignment with genes deposited in numerous databases, including the Gene Ontology (GO, parameter was default), Clusters of Orthologous Groups of Proteins (COG, evalue ≤ 1e^−5^), and Protein Families (Pfam, parameter was default) databases from the National Center for Biotechnology Information (NCBI), nonredundant protein (Nr, evalue ≤ 1e^−5^) database, UniProt/Swiss-Prot (evalue ≤ 1e^−5^) and Kyoto Encyclopedia of Genes and Genomes (KEGG, evalue ≤ 1e^−5^). Based on the Pathogen Host Interactions (PHI, evalue ≤ 1e^−5^) [61], Carbohydrate-Active enZYmes (CAZy, evalue ≤ 1e^−5^) [30] databases and database of Known Fungal Virulence Factors (DFVF, evalue ≤ 1e^−5^) [29], additional annotation was done. SignalP 4.0 (parameter was default) was used to predict secretory proteins [62]. In order to forecast secondary metabolic gene clusters, Antismash 4.1.0 (parameter was default) [63] was employed.

### Phylogenetic analyses

We constructed a phylogenetic tree using the maximum likelihood method with *Metschnikowia bicuspidata* and *Pichia kudriavzevii* (GCF003054445.1), *Ogataea angusta* (GCA019207455.1), *Brettanomyces bruxellensis* (GCF011074885.1), *Saccharomyces cerevisiae* (GCF000146045.2), *Saccharomyces kudriavzevii* (GCA000167075.2), *Kazachstania africana* (GCF000304475.1), *Candida tropicalis* (GCF000006335.3), *Candida albicans* (GCF000182965.3), *Candida parapsilosis* (GCF000182765.1), *Debaryomyces hansenii* (GCF000006445.2), *Candida auris* (GCA003013715.2), *Candida haemuloni* (GCA019332025.1), *Metschnikowia pulcherrima* (GCA004217705.1), *Candida intermedia* (GCA900106115.1), and *Clavispora lusitaniae* (GCA009498055.1). The genes in gene families with single copies in the whole genome of the sequenced species and reference species were used, and the evolutionary tree was constructed using IQTREE (Version 1.6.3) [64]. to study the evolutionary relationships between species. Based on the results of the clustering analysis of homologous gene families, a single copy of a homologous gene was selected for multisequence alignment and alignment quality control (using MUSCLE (Version 3.8.31) [65]. Software for sequence alignment and Gblocks (Version 0.91b) [66]. software for alignment quality control), and a phylogenetic tree was then constructed based on the single-copy gene method.

### Gene family analysis

We selected the genomes of five species (*Candida auris*, *Candida haemuloni*, *Metschnikowia pulcherrima*, *Candida intermedia*, *Clavispora lusitaniae*) showing similarity to the sample genomes and analysed gene families based on the BBH (bidirectional best-hit) standard (80% of the shortest protein sequences presented 40% amino acid similarity). The amino acid (or nucleotide) sequences of all species involved in the analysis were compared by using DIAMOND (Version 2.0.7) [67], similarity clustering was carried out by using OrthoMCL (Version 1.4) [68]. to obtain the list of homologous genes in clusters, and the species distribution of each protein cluster was counted.

## Supplementary Information

**Additional file 1: Figure S1. **Gene length distribution of *M. bicuspidata* strain MQ2101.

**Additional file 2: Figure S2. **The Venn diagram of annotated genes in COG/KOG, KEGG, NR and Swissprot databases.

**Additional file 3: Table S1.** The Nr functional annotation information.

**Additional file 4: Table S2. **The KOG functional annotation information.

**Additional file 5: Table S3. **The KEGG functional annotation information.

**Additional file 6: Table S4. **The GO functional annotation information.

**Additional file 7: Table S5. **The PHI genes annotation information.

**Additional file 8: Table S6. **The DFVF genes annotation information.

**Additional file 9: Table S7. **The CAZy genes annotation information.

**Additional file 10: Figure S3. **Major distribution of secretory proteins in MQ2101 by Gene Ontology analysis.

**Additional file 11: Figure S4. **Major distribution of specific genes in MQ2101 by Gene Ontology analysis.

## Data Availability

The datasets generated and/or analysed during the current study are available in the NCBI GenBank (https://www.ncbi.nlm.nih.gov/genbank/), the accession number of our submission is BioProject: PRJNA891789 or BioSample: SAMN31358899.
