# Supplementary figures and images for: Complete genome analysis of pathogenic Metschnikowia bicuspidata strain MQ2101 isolated from diseased ridgetail white prawn, Exopalaemon carinicauda
Source: BMC Microbiol. 2023 Apr 29;23:120. doi: 10.1186/s12866-023-02865-2 (PMC10148492; doi:10.1186/s12866-023-02865-2)

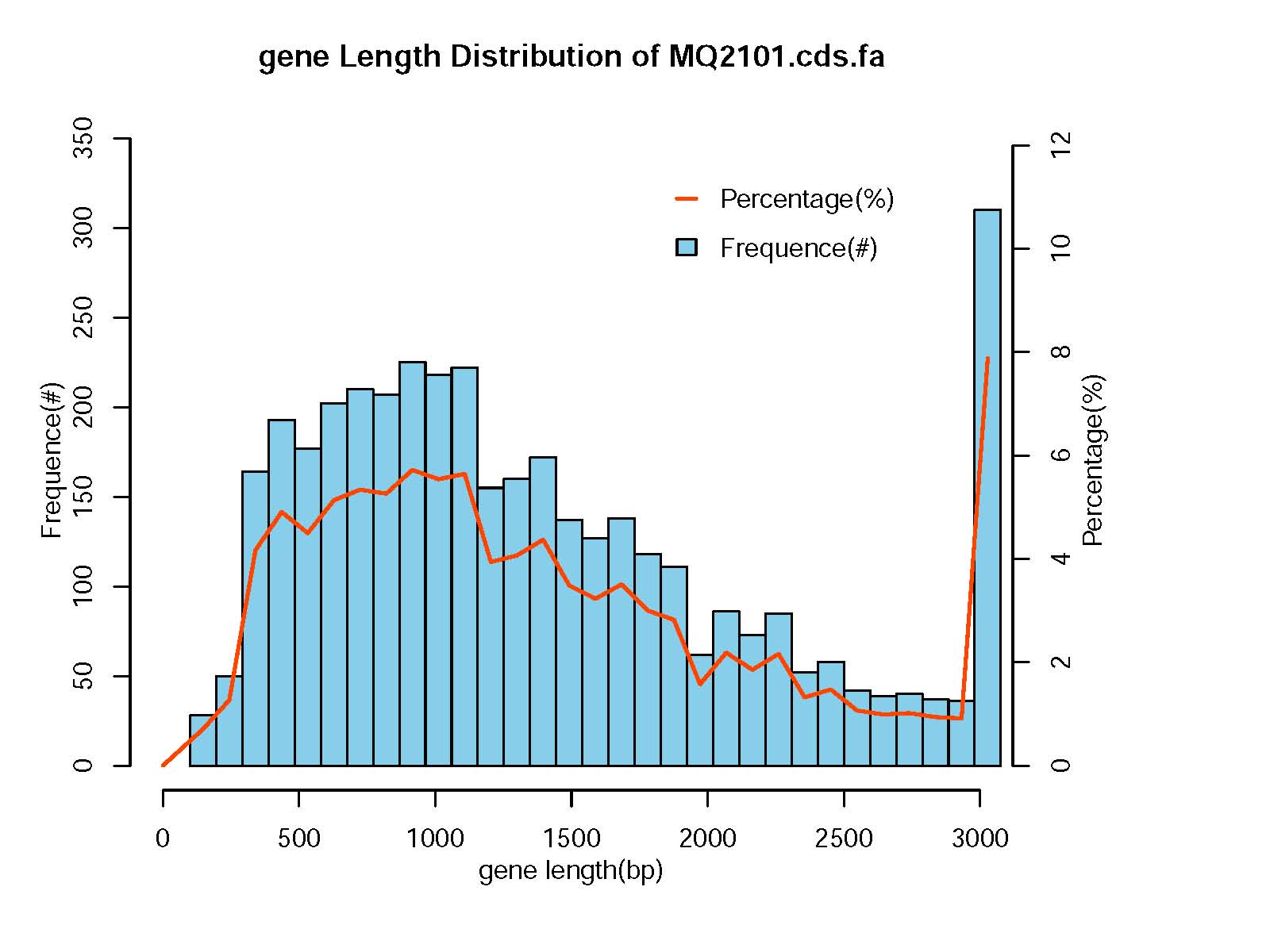


**Figure S1. Gene length distribution of *M. bicuspidata* strain MQ2101**

Supplement: Supplementary file 1 — Additional file 1: Figure S1. Gene length distribution of M. bicuspidata strain MQ2101. [file 12866_2023_2865_MOESM1_ESM.docx]

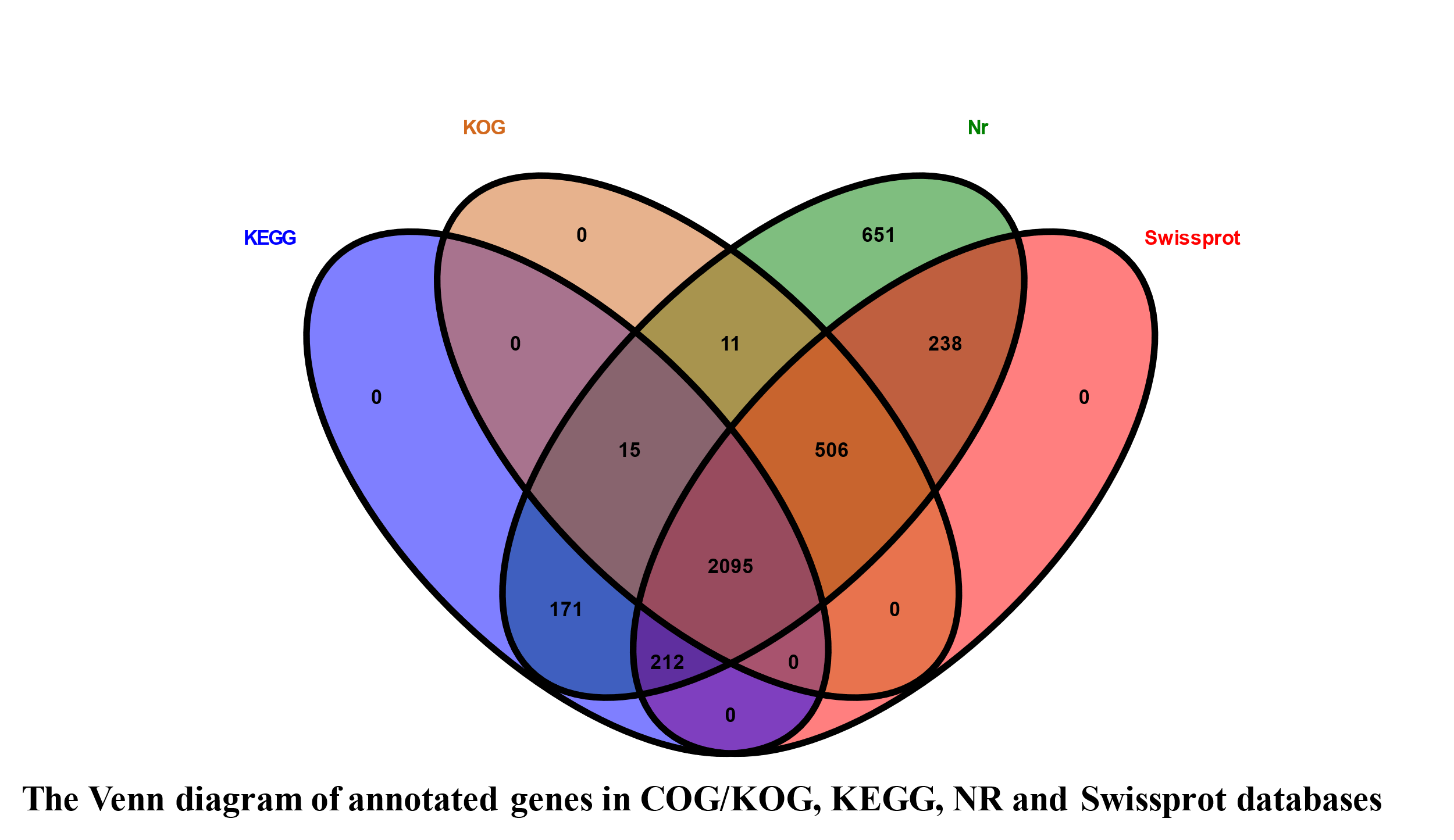


**Figure S2. The Venn diagram of annotated genes in COG/KOG, KEGG, NR and Swissprot databases.**

Supplement: Supplementary file 2 — Additional file 2: Figure S2. The Venn diagram of annotated genes in COG/KOG, KEGG, NR and Swissprot databases. [file 12866_2023_2865_MOESM2_ESM.docx]

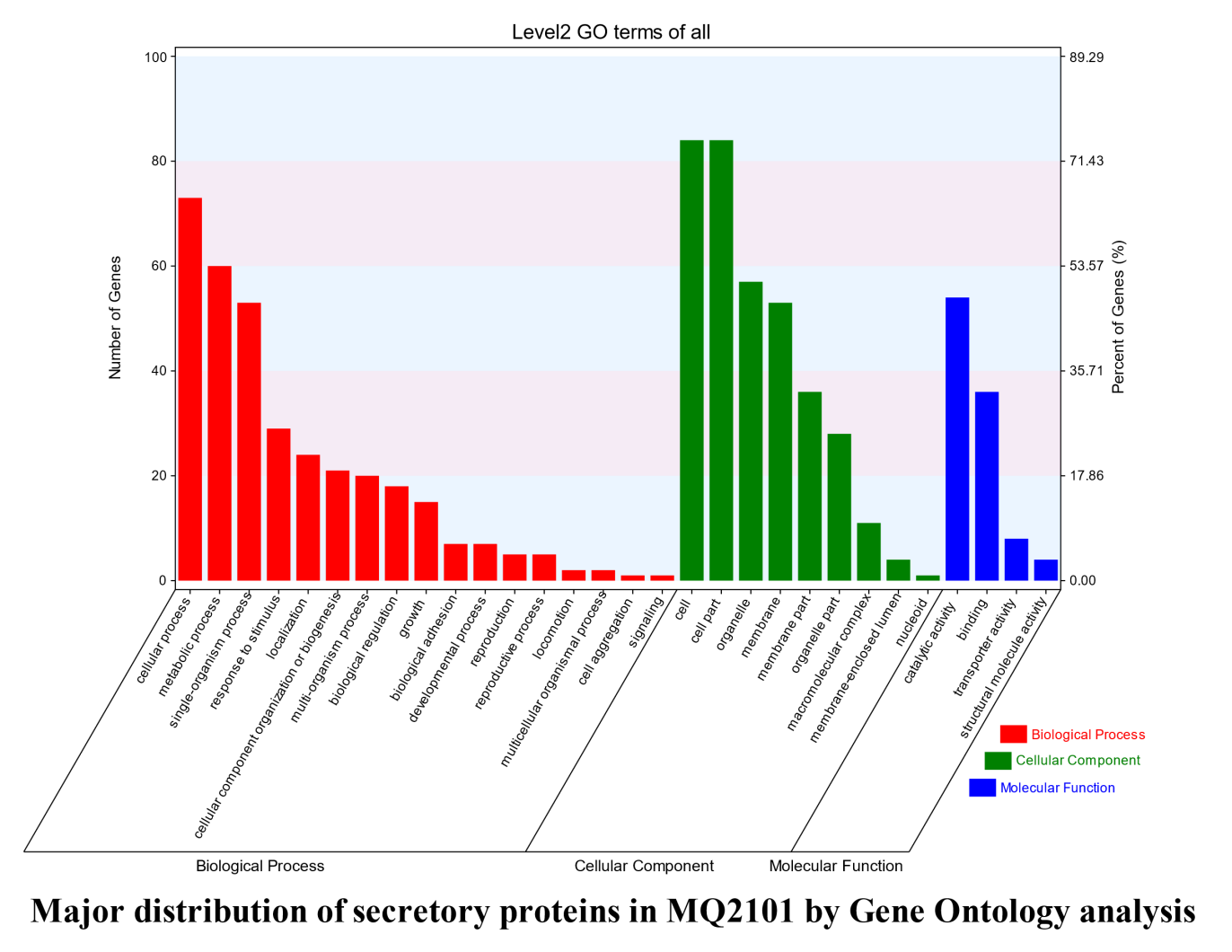


**Figure S3. Major distribution of secretory proteins in MQ2101 by Gene Ontology analysis.**

Supplement: Supplementary file 10 — Additional file 10: Figure S3. Major distribution of secretory proteins in MQ2101 by Gene Ontology analysis. [file 12866_2023_2865_MOESM10_ESM.docx]

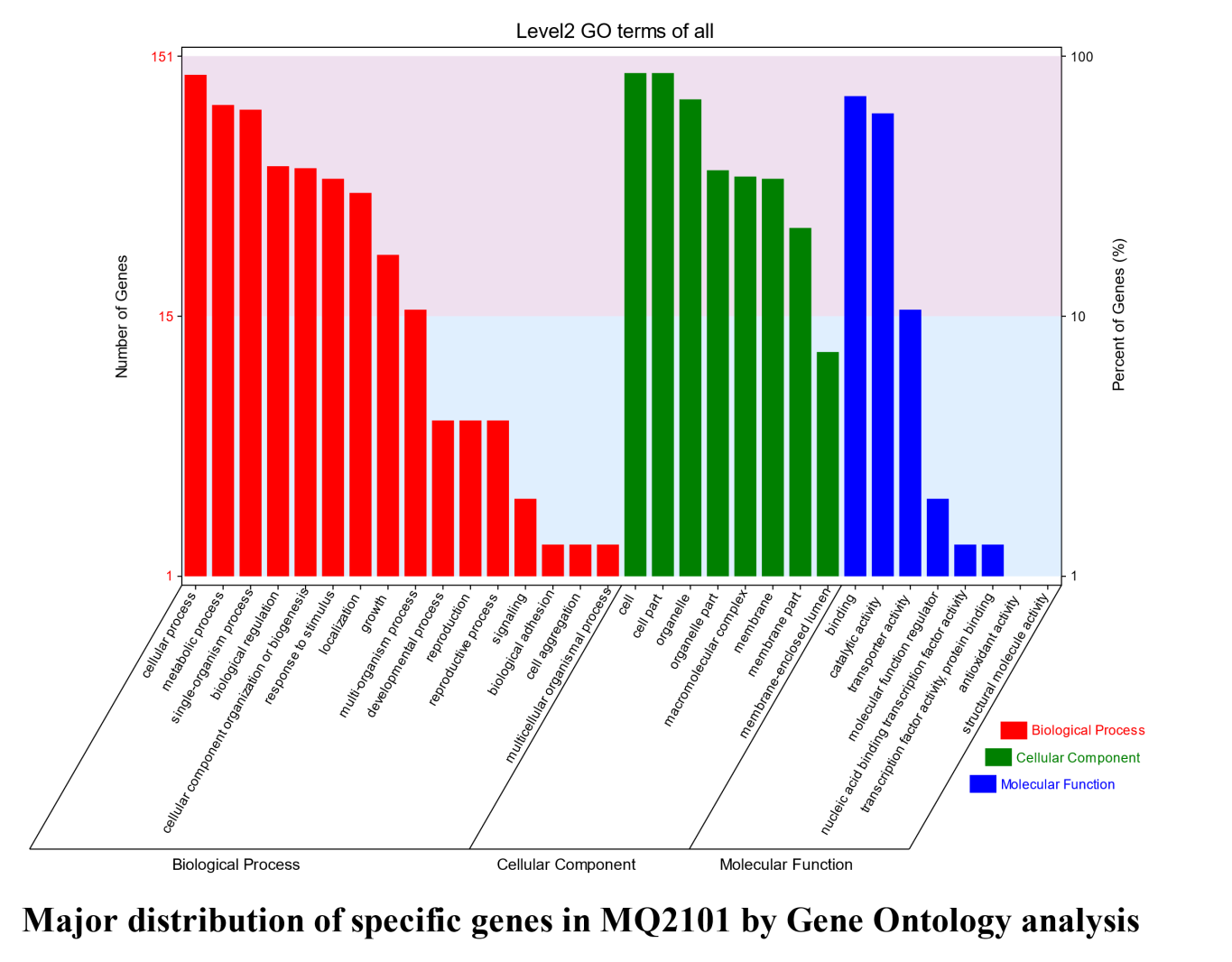


**Figure S4. Major distribution of specific genes in MQ2101 by Gene Ontology analysis.**

Supplement: Supplementary file 11 — Additional file 11: Figure S4. Major distribution of specific genes in MQ2101 by Gene Ontology analysis. [file 12866_2023_2865_MOESM11_ESM.docx]
